# Associations between quality of life and socioeconomic factors, functional impairments and dissatisfaction with received information and home-care services among survivors living at home two years after stroke onset

**DOI:** 10.1186/1471-2377-14-92

**Published:** 2014-04-28

**Authors:** Michèle Baumann, Etienne Le Bihan, Kénora Chau, Nearkasen Chau

**Affiliations:** 1 Medical sociologist, Research Unit INSIDE, Institute Health & Behaviour, University of Luxembourg, L-7201 Walferdange, Luxembourg; 2 Statistician, Research Unit INSIDE, Institute Health & Behaviour, University of Luxembourg, L-7201 Walferdange, Luxembourg; 3 Physician and epidemiologist, Lorraine University, Faculty of Medicine, Service of General Medicine, F-54505 Vandoeuvre-lès-Nancy, France; 4 Epidemiologist, INSERM U669, Univ Paris-Sud and Univ Paris Descartes, UMR-S0669 Paris, France

**Keywords:** Newcastle Stroke-Specific Quality of Life, Newsqol, Quality of life, Dissatisfaction, Home-care services, Information, Post-stroke

## Abstract

**Background:**

Quality of life (QoL) assessment is important when monitoring over time the recovery of stroke-survivors living at home. This study explores the associations between QoL and socioeconomic factors, functional impairments and self-reported dissatisfaction with received information and home-care services among survivors two years after stroke onset. This problem remains partially addressed though optimal information and services may improve survivors’ QoL.

**Methods:**

Stroke-survivors admitted to all hospitals in Luxembourg 18 months or more previously were identified using the only care-expenditure-reimbursement national system database. The clinical diagnosis was confirmed. Ninety four patients aged 65 years and living at home were interviewed to gather socioeconomic characteristics, functional impairments, dissatisfaction with information and home-care services, and QoL (using the Newcastle Stroke-Specific QoL, newsqol) assessing 11 domains. Data were analyzed using multiple linear regression models.

**Results:**

About 50% of survivors had low education and lower income. Functional impairments were common: sensory (45%), motor (35%), memory (32%), language (31%), and vision (20%). Survivors with education (<12th grade) or lower income had low values for most newsqol domains (sex-age-adjusted regression coefficient saRC, i.e. mean difference, between -23 and -8). Patients who were working had better values for pain, mental feelings and sleep domains than did retired people (saRC between -3.9 and 4.2). Various functional impairments were associated with markedly low values of nearly all domains (saRC between -33.5 and -7.5) and motor, language, memory and sensory impairments had the highest impact. The survivors’ perceived QoL was markedly low, especially for the domains of interpersonal relationship, sleep, cognition, mental feelings, and pain. Various QoL domains were strongly related to dissatisfaction with information about stroke and its consequences/changes over time, accuracy of information obtained, help received, coordination between services, and the possibility of receiving help when necessary (saRC reaching -30).

**Conclusions:**

Stroke-survivors had major alterations in QoL that reflected depressive symptoms, which should be appropriately treated. These findings may help with the development of public policies aiming at improving QoL among stroke survivors. The newsqol could be used routinely to measure the recovery of survivors over time and their needs in terms of information, help and care services.

## Background

Cerebrovascular diseases are a public health concern in the European Union (EU) because they are common and may result in a wide range of severe permanent impairments and associated functional limitations in daily living activities, and because they represent the third leading cause of mortality [1-5]. Cerebrovascular diseases are also a public health issue in Luxembourg, one of the smallest EU countries [2]. Stroke-survivors may have low quality of life (QoL), may have to leave their jobs and may face a number of new problems: unknown severity of stroke, its health consequences, its evolution over time, fear of aggravation, a change in socioeconomic situations, need for care and information about stroke and its consequences, and available help/services [4,6]. These problems may be more pronounced among survivors with more severe stroke that results in multiple disabilities and mental difficulties such as depressive symptoms, and low capability for mobility, self-care, living environment control, and exchanges with others [4]. Therefore, it is important to monitor the QoL of stroke-survivors using the Newcastle Stroke-Specific QoL Measure (newsqol) as it is constructed to evaluate a number of relevant domains including mobility, self-care, pain, vision, cognition, communication, mental feelings, interpersonal relationships, emotion, sleep problems, and fatigue [7]. The newsqol is recommended for assessing the recovery of survivors [8]. It has become a relevant measure to evaluate the functional impairments resulting from stroke and their consequences, as well as care and information from social and medical services needed by the survivors. Such assessment is important for patients during hospitalization, but even more so for survivors living at home. Alteration in issues such as newsqol domains prompts concern about the future in terms of consequences of stroke, daily living activities, desire for improvement and fear of aggravation [9]. Some newsqol domains reflect depressive symptoms, which are associated with injury, disability and premature death [10,11]. One study reported a risk of depression after stroke [12].

The situation of stroke-survivors is difficult because the level of stroke at onset is often not well established and the evolution of morbidity over time is often unknown, even for carers. The information about stroke given to patients and their families may be limited and uncertain depending on the subsequent changes expected and observed. This situation could leave the survivors and their families uncertain about the future and the care, help and information they may need, as well as about what services are available, who to contact and how to obtain appropriate help, and lacking confidence in the information received. For survivors living at home, the information and help received may be diverse because the level of stroke and associated morbidity may remain unclear over time. Furthermore, because stroke and associated disabilities affect capacity to work, many survivors have to leave their jobs (to retire or become unemployed), which may result in low socioeconomic resources. These issues are likely to be more pronounced among those individuals in lower socioeconomic groups, those with a lower educational level and income, and possibly those with more severe stroke and comorbidity [13,14]. The individuals in lower socioeconomic groups or with a lower educational level may have reduced awareness of the benefits of prevention. It would be of interest to evaluate the ways in which socioeconomic characteristics may impact on various newsqol domains. This raises the problem of identification of most vulnerable people, for whom care services, help, information and prevention should be a priority. Note that reduced QoL has been found to be related to demographic factors and health disorders such as sex, education, comorbidity, and psychological disorders [4]. QoL is shaped by diseases and functional impairments, individual choices and behaviours, but the surrounding environment and the relevance of help and information received are also major determinants [15]. It has been recognised that medical and community support may not be equitably provided, and that socioeconomic status plays a strong role in access to services [16]. The impact of socioeconomic deprivation may increase among survivors, because of functional limitations and reduced daily living activities, leisure and occupational activities [17]. Because stroke is more common among older people, isolation and poor family support, functional impairments, disabilities and reduction in daily living activities [18] associated with ageing should also be considered.

The social services and health systems must ensure that help and information are available and accessible to all stroke-survivors whatever their socioeconomic status [19,20]. The evaluation of newsqol domains and their association with various functional impairments and socioeconomic features is useful for stroke-survivors. It would identify specific functional impairments to be treated and what newsqol domains are reduced, as well as pinpointing the most vulnerable socioeconomic groups. Knowledge of these issues may help stroke-survivors and their families to better understand the situation they find themselves in and facilitate efforts to find care, help and information. It may also help social and medical carers to establish the services, help and information needed by the subjects, especially rehabilitation, and to discuss issues with them in a patient-centred approach, which may reduce the stress of both patients and carers, and improve their cooperation [21-23].

The tasks of social and medical carers who intervene at home may not be easy. Indeed, they may not have received the training they need to face such situations, and the care services and help that they provide may lack coordination. The time available for patients is generally limited. Stroke-survivors may therefore be more exposed to stress, mental difficulties, and an alteration in their QoL, particularly those with more severe stoke. Studies have shown a lack of satisfaction among stroke patients with the provision received, the quality of materials provided, and availability of stroke-related information [24]. Further studies are therefore needed to assess the relationships of dissatisfaction with received information and home-care services with newsqol domains among survivors living at home, after stroke onset. A two-year time lapse may be considered appropriate as the consequences of stroke may be relatively stable by that time. Such studies could provide knowledge to improve the QoL of stroke-survivors, but would also elucidate the training that society has to provide to carers to help them to achieve their tasks. Satisfaction with care received may somewhat reflect the quality of care and increasing it could reduce stress of both patients and caregivers and improve patients’ QoL [25].

Satisfaction of stroke-survivors living at home is increasingly recognized as a key focus for policies aimed at improving the quality of home-care. It may be useful to explore the expectations and requests of patients, the home-care provided, and its impact on patients’ QoL [26,27]. This issue has remained partially addressed while studies have shown that access to care services varies substantially according to location and income [28]. Appropriate home-care services should improve the patients’ QoL but also foster motivation and reassurance, and provide necessary information.

The present study aimed to explore, among survivors living at home two years after stroke onset: (1) the associations between newsqol and socioeconomic characteristics and functional impairments; and (2) the associations between newsqol and self-reported dissatisfaction with various information and home-care services received.

## Methods

### Study sample and design

The sample studied included all stroke-survivors (797 patients) admitted to all hospitals in Luxembourg, 18 months or more before the day of survey as identified in the ‘*Inspection Générale de la Sécurité Sociale*’ (the only national system for care expenditure reimbursement). The database identified all treated stroke patients, and their survival was determined from the Civil Status Registry.

The inclusion criteria were: (a) living in Luxembourg at stroke onset; (b) a diagnosis of stroke (ICD-10 codes I60, I61, I62, I63, I64, and G46); (c) living at home in Luxembourg (not in an institution) 2 years after stroke onset; (d) understanding Luxembourgish, Portuguese, French or German, for the face-to-face interview, by patient or primary caregiver (one case excluded); and (e) valid home address (11 cases excluded).

A total of 374 patients fulfilled these criteria. Aphasic patients were also included (researchers had been trained to communicate with them), but patients with transitory ischemic attacks were excluded.

A letter was then sent to them to explain the aims of the national survey, obtain their agreement to consult their records, and to obtain permission for a researcher to visit their home. Clinical diagnosis of cerebrovascular disease was confirmed by the medical investigator. After receiving signed informed consent, the research team telephoned (up to five attempts) to make an appointment at the patient’s home and conducted the face-to-face interviews supported by a questionnaire. Of the 374 subjects contacted, 94 participated (participation rate 25%).

The face-to-face interview gathered demographic and socioeconomic data: sex, age, live in couple vs. not, nationality (Luxembourgish vs. other); educational level (under 12^th^ grade vs. higher), occupation at the time of stroke onset (never employed, manual worker, employee/intermediate professional/technician, manager and professional), current socio-occupational category (working, retired, or unemployed), income (<3000€/per month, representing three times the minimum wage, vs. higher) [29], and residence municipality (Luxembourg City; 10 communes of more than 7500 inhabitants; other municipalities) [29]. The interviewer then measured QoL (using the Newcastle Stroke-Specific QoL Measure, newsqol) [7], functional impairments (with American Heart Association Stroke Outcome Classification, AHA.SOC) [30], and dissatisfaction with information and home-care services [31,32]. As Luxembourg is multilingual, the questionnaire was in four official languages. Most of the instruments were already available in French or English. The German, Portuguese and Luxembourgish versions were translated, back-translated, and proofread by native-speaking professional translators.

The protocol was approved by the National Committee of Research Ethics (NCRE) and the Committee for Data Protection of Luxembourg. The NCRE did not authorize us to contact the 242 patients and their families who failed to respond.

### Measures

#### QoL

QoL was evaluated using the Newcastle Stroke-Specific Quality of Life Measure (newsqol) [7], which included 11 multi-item subscales measuring mobility, self-care, pain, cognition, vision, communication, mental feelings, interpersonal relationships, emotion, sleep and fatigue domains (Additional file 1). Responses for each item of each domain, ranged from 1 (worst) to 4 (best). The score of each domain was defined as the sum of the responses, and then modified to range from 0 (lowest) to 100 (best).

#### Functional impairments

The American Heart Association Stroke Outcome Classification (AHA.SOC) [30] was utilized. It is a validated instrument measuring neurologic functional impairments associated with stroke, which included motor, vision, sensory, language, and memory impairments, and also incontinence and personality disorders, which are considered to be residual disabilities (Additional file 2).

#### Dissatisfaction with information and home-care services

**Figure 1:**
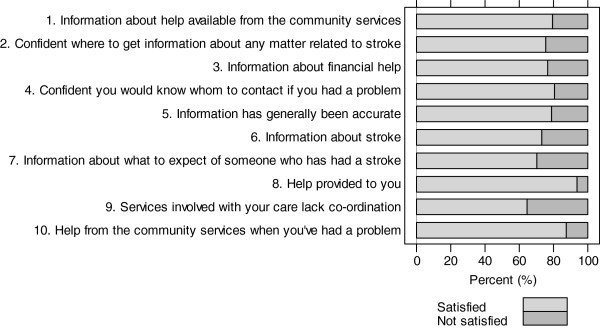
Distribution of the answer to the items of dissatisfaction with information and home-care received (%).

Dissatisfaction with perceived information and home-care services received was measured for ten issues as shown in Figure 1[31,32]. Information was gathered about community support, coordination and appropriateness of services, characteristics of stroke, speed of change, listening and being heard, problem management and accuracy of information. Responses for each item ranged from: completely agree, to agree, disagree or completely disagree.

### Statistical analysis

The outcome variables were the 11 newsqol domains considered as continuous variables. The correlation between each domain and socioeconomic factors (considered as qualitative variables) was assessed using multiple linear regression models to compute the sex-age-adjusted regression coefficient (saRC) and standard error (SE). Sex and age were taken into account because they are potential confounders for the issues investigated. The saRC was also used to evaluate the association of each newsqol domain with each category of functional impairment (considered as binary variable), as well as with each item measuring dissatisfaction with information and home-care received (also considered as binary variable). To assess the relationships two by two between various newsqol domains we used the Pearson correlation coefficients. The analyses were performed using the R program.

## Results

### Characteristics of stroke survivors

As Table 1 shows, the mean age of survivors was 65.5 years; men represented 55% of subjects; 75% were living in a couple and 74% were Luxembourgish. Half had a low-level education. At the time of stroke onset, a third had been manual workers, and another third employees, intermediate professionals or technicians. At the time of the survey, most survivors were retired or unemployed; 46% of them had a lower income. Each survivor suffered from a mean of 2 functional impairments, the main being sensory (45%). They had markedly low QoL, especially for the interpersonal relationship, sleep, cognition, mental feelings, and pain domains.

**Table 1:** Socioeconomic characteristics of stroke-survivors (N = 94): mean (standard deviation (SD)) or %

	**Mean (SD) or %**
Age (mean (SD)) (year)	65.5 (14.4)
Men	55
Luxembourgish nationality	74
Living in couple	75
Low-level educational (< 12th grade)	53
*Current occupational status*	
Working	16
At home without activity	27
Retired	57
*Occupation at the time of the stroke*^a^	
Never employed	16
Manual worker	32
Employee, intermediate professional and technician	31
Manager and professional	21
Lower income	46
*Town of residence*	
Luxembourg city	10
Most populous towns (more than 7,500 inhabitants, 10 towns)	28
Other areas	63
*Current neurologic impairments AHA.SOC*[28]	
Functional impairments^b^	
Motor	35
Vision	20
Sensory	45
Language	31
Memory	32
Incontinence	12
Personality disorders	17
*Quality of life (score measured with the Newsqol*[27]*) (0-100)*^c^	
Mobility	82.5 (23.2)
Self-care	86.6 (22.6)
Pain	78.9 (27.4)
Cognition	76.3 (23.8)
Vision	85.0 (23.9)
Communication	81.3 (21.9)
Mental feelings	77.2 (24.8)
Interpersonal relationships	89.0 (15.4)
Emotion	72.5 (25.2)
Sleep	75.1 (23.9)
Fatigue	79.5 (24.6)

^a^For unemployed, retired people and those in vocational training at the time of stroke, the last occupational activity was considered.

^b^The sum of the percentages was 192%, i.e. the mean number was 2 per subject.

^c^The score of each item was a continuous variable with value between for the newsqol domain (0 = worse quality of life; 100 best quality of life).

### Dissatisfaction with information and home-care services received

Figure 1 shows that dissatisfaction was relatively common (up to about 40%) and concerned mainly lack of co-ordination of care services, information about stroke, its consequences and its evolution over time, and accuracy of information received. A high proportion of survivors reported a lack of knowledge about how, where and who to contact to obtain necessary information, financial help and community services.

### Associations of newsqol with socioeconomic factors and functional impairments

Table 2 shows that subjects with low-level education (under 12^th^ grade) had low values for the newsqol domains of pain and emotion (saRC -20.8 and -19.3, respectively). In addition, various functional impairments were associated with markedly low values for nearly all domains (saRC between -33.5 and -7.5), and that motor, language, memory and sensory impairments had the greatest impact on newsqol.

**Table 2:** Associations of newsqol domains with impaired functions and socioeconomic factors among stroke-survivors: regression coefficient (standard error SE)

	**Newsqol domains**										
	**Mobility**	**Self-care**	**Pain**	**Cognition**	**Vision**	**Communication**	**Mental feelings**	**Interpersonal relationships**	**Emotion**	**Sleep**	**Fatigue**
**Sex-age-adjusted regression coefficient**											
**Socioeconomic factor**^a^											
Luxembourgish nationality	**-**	**-**	14.2^*^ (6.9)	**-**	17.0^**^ (5.5)	**-**	**-**	**-**	**-**	**-**	**-**
Low-level education (<12^th^ grade)	-15.6^**^ (4.9)	-14.3^**^ (4.9)	-20.8^***^ (6.0)	**-**	**-**	-12.7^*^ (4.9)	-15.7^**^ (5.6)	-10.7^**^ (3.4)	-19.3^***^ (5.5)	-10.6^*^ (5.3)	**-**
*Current occupational status (vs.* retired people)											
Working	**-**	**-**	32.6^**^ (11.5)	**-**	**-**	**-**	27.1^**^ (10.3)	**-**	**-**	17.7^*^ (9.8)	**-**
At home without activity	**-**	**-**	4.2^**^ (11.3)	**-**	**-**	**-**	1.5^**^ (10.1)	**-**	**-**	-3.9^*^ (9.7)	**-**
Lower income	-12.2^*^ (5.1)	-12.4^*^ (5.4)	-22.9^**^ (7.0)	**-**	**-**	**-**	-17.9^**^ (6.2)	-8.4^*^ (4.1)	-20.8^**^ (6.2)	-13.1^*^ (5.7)	**-**
**Functional impairments**											
Motor	-24.2^***^ (4.2)	-23.3^***^ (4.2)	-25.5^***^ (5.4)	-14.1^**^ (5.1)	-11.5^*^ (4.9)	-15.7^***^ (4.5)	-26.8^***^ (4.7)	-7.5^*^ (3.3)	-16.8^**^ (5.3)	-14.1^**^ (5.1)	-16.6^**^ (5.1)
Vision	-13.5^*^ (6.1)	**-**	**-**	-14.1^*^ (6.5)	-33.5^***^ (5.3)	**-**	**-**	-9.2^*^ (4.2)	-18.3^**^ (6.8)	**-**	**-**
Sensory	-15.9^***^ (4.7)	-16.9^***^ (4.6)	-29.1^***^ (5.2)	-16.4^**^ (5.0)	**-**	-16.9^***^ (4.5)	-14.9^**^ (5.3)	-7.6^*^ (3.3)	-19.7^***^ (5.2)	-18.6^***^ (4.9)	-17.8^***^ (5.0)
Language	-21.3^***^ (4.8)	-21.5^***^ (4.7)	-22.2^***^ (6.1)	-24.4^***^ (5.1)	-23.1^***^ (4.9)	-24.9^***^ (4.5)	-23.8^***^ (5.4)	-10.2^**^ (3.5)	-18.8^**^ (5.7)	-20.3^***^ (5.3)	-26.7^***^ (5.0)
Memory	-15.5^**^ (4.9)	-12.0^*^ (5.0)	-28.8^***^ (5.6)	-26.4^***^ (4.8)	-11.9^*^ (5.1)	-14.3^**^ (4.9)	-23.6^***^ (5.2)	-10.7^**^ (3.4)	-22.8^***^ (5.4)	-20.0^***^ (5.2)	-19.4^***^ (5.3)
Incontinence	-31.8^***^ (8.2)	-25.3^**^ (8.3)	-37.4^***^ (10.1)	**-**	**-**	-22.5^**^ (8.4)	-23.8^*^ (9.5)	**-**	-20.9^*^ (9.8)	**-**	-21.3^*^ (9.3)
Personality disorders	**-**	**-**	**-**	-21.5^**^ (6.7)	**-**	**-**	**-**	-15.1^***^ (4.3)	-15.2^*^ (7.4)	-15.9^*^ (7.0)	-24.0^***^ (6.8)

^*^p <0.05; ^**^p < 0.01; ^***^p < 0.001.

“-”: Non-significant regression coefficient (p > 0.05).

^a^Living in couple, occupation at stroke onset, current occupational status, and town of residence were not related to at least one newsqol domain. They are not presented to alleviate the Table.

Note: No significant regression coefficients were found for all socioeconomic factors (see Table 1) in full models including all socioeconomic factors and all impaired functions. These factors are not presented in the Table.

### Concomitant presence of various newsqol domains

As shown by the Pearson correlation coefficients in Table 3, strong two-by-two interdependence was observed between most newsqol domains, i.e. their presence was often concomitant. They were particularly high for emotion, sleep, cognition, mental feelings, pain and fatigue (which are considered as depressive symptoms in the literature) [25]. Mobility was also strongly related to self-care, mental feelings, communication, and emotion.

**Table 3:** Pearson correlation coefficients between various newsqol domains

	**Mobility**	**Self-care**	**Pain**	**Cognition**	**Vision**	**Communication**	**Mental feelings**	**Relationships**	**Emotion**	**Sleep**
Self-care	0.87***									
Pain	0.45***	0.51***								
Cognition	0.48***	0.44***	0.62***							
Vision	0.18	0.06	0.37***	0.52***						
Communication	0.63***	0.57***	0.40***	0.57***	0.39***					
Mental feelings	0.74***	0.71***	0.58***	0.64***	0.31**	0.59***				
Relationships	0.34**	0.33**	0.40***	0.45***	0.16	0.38***	0.63***			
Emotion	0.58***	0.54***	0.53***	0.61***	0.26*	0.51***	0.81***	0.62***		
Sleep	0.45***	0.45***	0.54***	0.61***	0.37***	0.47***	0.58***	0.43***	0.62***	
Fatigue	0.53***	0.49***	0.40***	0.63***	0.25*	0.62***	0.68***	0.51***	0.60***	0.53***

*p <0.05; **p < 0.01; ***p < 0.001.

### Associations between newsqol and dissatisfaction with information and home-care services

Table 4 reveals that survivors who were dissatisfied with information and home-care received had rather low values for several newsqol domains (saRC between -30.3 and -7.7). Dissatisfaction with accuracy of information received, information about stroke and its consequences/change over time; help received, lack of coordination between services, and possibility of receiving help were related with low values for most domains (saRC between -30.5 and -7.7). Dissatisfaction with the possibility of receiving help was also associated with mobility and self-care (saRC -21.9 and -19.9, respectively). Dissatisfaction with information about consequences/change over time of stroke linked with the vision domain (saRC -12.6). Dissatisfaction with information received about help correlated with mental feelings, interpersonal relationships, emotion, and fatigue (saRC -22.7, -16.2, -19.7 and -14.3, respectively). Being confident about where to get information about stroke, looking after someone who has had a stroke if needed, or about financial help, and being confident about who to contact from community services if needed were slightly associated with communication, mental feelings and interpersonal relationships only (saRC between -15.2 and -10.4).

**Table 4:** Associations between newsqol domains and dissatisfaction with information and home-care received among stroke-survivors: regression coefficient (standard error SE)

	**Newsqol domains**^**a**^									
	**Mobility**	**Self-care**	**Pain**	**Vision**	**Communication**	**Mental feelings**	**Interpersonal relationships**	**Emotion**	**Sleep**	**Fatigue**
**Sex-age-adjusted regression coefficient**										
1-Information you have been given about help available from the community services for you (include health, social and voluntary services)	-	-	-	-	-	-22.7^***^ (6.3)	-16.2^***^ (3.8)	-19.7^**^ (6.6)	-	-14.3^*^ (6.3)
2- Confident you would know where to get information about any matter related to stroke or looking after someone who has had a stroke if you needed it	-	-	-	-	-	-15.2^*^ (6.1)	-	-	-	-
3- Information you have been given about financial help you might be entitled to (including benefits)	-	-	-	-	-	-15.1^*^ (6.9)	-10.4^*^ (4.2)	-	-	-
4- Confident you would know whom to contact from the community services if you had a problem	-	-	-	-	-12.2^*^ (5.8)	-	-	-	-	-
5- Information you have received has generally been accurate	-	-	-15.0^*^ (7.5)	-	-	-	-9.8^**^ (3.6)	-15.5^*^ (6.7)	-	-26.6^*^ (7.2)
6- Information you have been given about stroke	-	-	-19.8^**^ (6.5)	-	-11.5^*^ (5.3)	-19.3^**^ (5.9)	-7.7^*^ (3.8)	-14.8^*^ (6.1)	-16.9^**^ (5.2)	-
7- Information you have been given about what to expect of someone who has had a stroke	-	-	-14.6^*^ (6.4)	-12.6^*^ (5.3)	-10.2^*^ (5.1)	-16.2^**^ (5.7)	-	-	-16.2^**^ (5.4)	-
8- Help provided to you (include in this help to allow you time to do what you want to do and time off)	-	-	-28.3^*^ (13.0)	-	-25.5^*^ (10.4)	-24.3^*^ (11.9)	-17.9^*^ (7.1)	-30.5^*^ (12.0)	-28.3^**^ (10.3)	-
9- Services involved with your care lack coordination or don’t work together	-	-	-	-	-15.0^**^ (5.0)	-	-	-	-	-
10- Help you have received from the community services when you have had a problem	-21.9^**^ (6.7)	-19.9^**^ (6.7)	-28.6^**^ (9.2)	-	-25.4^***^ (7.0)	-30.3^***^ (7.9)	-14.1^**^ (4.6)	-24.8^**^ (8.4)	-26.6^***^ (7.2)	-16.6^*^ (8.1)

^*^p <0.05; ^**^p < 0.01; ^***^p < 0.001.

“-”: Non-significant regression coefficient (p > 0.05).

^a^The cognition domain was not associated with any dissatisfaction item.

## Discussion

The present study demonstrates that, two years after stroke onset, the survivors’ perceived QoL was markedly low for the following domains: emotion, sleep, cognition, communication, mobility, mental feelings, pain and fatigue (which are generally known as depressive symptoms) [11] and the alterations were strongly associated with dissatisfaction with information and help received, lack of coordination between services, and concerns about the possibility of receiving help when necessary. The low QoL of survivors reflected in fact the consequences of a wide range of functional impairments, and the most vulnerable survivors were those with low-level education, lower income, or who stopped working. A small difference was observed between Luxembourgers and other nationalities.

We found that, two years after stroke onset, a high proportion of survivors suffered from a number of neurologic functional impairments of which motor, language, memory and sensory impairments had the greatest impact on nearly all newsqol domains. It should be noted that the mean number of functional impairments was two and that various newsqol domains were strongly interdependent, especially those representing depressive symptoms (emotion, sleep, cognition, communication, mobility, mental feelings, pain and fatigue). So, a functional impairment which may alter one of these domains was likely to alter the other domains as well. The stroke-survivors may be exposed to depressive symptoms when their impairments severely affect several domains of QoL. They may see this as reflecting the severity of stroke. One study showed that functional vision loss was associated with depression [33]. Depressive symptoms are known to reduce physical, mental and cognitive abilities, and increase the risk of injuries and premature death [10,11,34]. As a consequence, they may aggravate the situation of patients and favour injuries and long-term premature death. For each subject, the functional impairments and their consequences for QoL should also be globally evaluated by considering their possible cumulative effect. Multiple functional impairments and multiple low QoL domains may increase the dependency of subjects and the need for technical, financial and personal aids. For older people, who were the most represented, the issues are likely to be more complex because of ageing and associated lower income and poor social support [11].

Our study reports that motor, language and memory impairments were those most correlated with low newsqol domains. Considered alone, language impairment, particularly for aphasia patients, may have a profound impact on QoL at a personal level and in its social components [35]. Speech loss may be associated with extreme emotional reactions and isolation and thereby with poor social relationships. Inability to express oneself during social exchanges/relationships affects self-esteem and induces feelings of humiliation [36], which could limit subsequent exchanges with others and indeed all activities that involve speaking. Motor impairment was, not unexpectedly, correlated with mobility and self-care, but our study shows that it was also linked with mental difficulties including depressive symptoms, and especially pain and mental feelings. Memory impairment was mainly related with low QoL in cognition, pain, mental feelings, emotion, sleep and fatigue. Memory loss meant here not only failing to remember things, but also perceived losses in the ability to concentrate, think, solve problems, and make decisions. The efforts that subjects had to make every day could be perceived as a new and strange source of frustration [37]. Reduced memory may lead patients to lose sense of context and perspective of time and may result in a perception of being distant from those around them [37-40]. Memory impairment may therefore also be correlated with the QoL domains reflecting depressive symptoms. Because depression is one of the most prevalent mental disorders in developed countries, a growing contributor to the general burden of disease, and may become the most frequent cause of disability worldwide by 2030 [10,11,28,34,41], and because depressive symptoms were here common two years after stroke onset, our results raise the question of whether they had been appropriately treated. Moreover, a reduction in depressive symptoms means a reduction in injuries (especially work injuries and falls among the elderly), particularly among individuals with lower physical capability, and also a reduction in premature deaths [10,11,34]. It may be indicated that about half of people suffering from psychiatric disorders do not receive proper treatment, even though it can effectively decrease symptom levels and reduce the risk of persistence [42-44]. One study in France stated that 60% of individuals who have depression seek medical treatment [45].

In this study, a small difference in newsqol was observed between Luxembourgers and other nationalities (for pain and vision domains only) while low-level education, lower income and becoming inactive (retired or unemployed) were associated with mobility, self-care and a number of domains concerning depressive symptoms. This may be attributed to possible more severe stroke and to more mental difficulties due to health and socioeconomic problems. These findings identify the subjects most at risk and to whom particular attention should be paid in terms of prevention and intervention. The financial and psychological instability that accompanies negative life events may reveal or revive latent weaknesses that otherwise would not appear and would not affect health. The effects of stroke would be amplified among subjects with socioeconomic disadvantages [46]. Our results are in accord with those of another study, which reported that most stroke-survivors had low QoL, a greater prevalence of stress and depression, more economic burden, and more change in social relationships than the general population [47]. Our findings have to be put into the socioeconomic context of Luxembourg, which is one of the smallest European countries (502,500 inhabitants, area 2600 km^2^) and has a high gross domestic product per inhabitant [48]. In our study, most stroke-survivors were Luxembourgers, inactive (retired or unemployed) and had over 3000€/per month. Luxembourgers have public or private resources, and services are principally professional. The distances between the population and services are short, and healthcare is geographically accessible to the whole population. The indicator of the quality of the National Health Service is 7.4/10 for Luxembourg vs. 6.2/10 for the EU-15 and 6.1/10 for the EU-27 (2007) [20].

Importantly, the present study demonstrates that the newsqol domains reflecting depressive symptoms (except the cognition domain) were the most related with dissatisfaction with information and home-care received. This was not surprising because those newsqol domains were strongly related to various functional impairments, which were rather long-lasting as they persisted two years after stroke onset. Our study reveals that a lack of information about stroke and its consequences/change over time, and a lack of accuracy of information received, help received, coordination between services, and of the possibility of receiving help when necessary were the most associated with the previous newsqol domains. There may thus be a true need to understand the disease, to know what consequences can be expected, to be offered good coordination between services, and knowledge about how to find necessary information. Being continuously dissatisfied was found to be correlated with depressive symptoms. However, the relationships may be bilateral. Indeed, people with depressive symptoms, and thus with lower physical and mental capabilities, may perceive the information and home-care received less positively. It should be noted that the issues are of more concern to people with low-level education, lower income, and who left their jobs after the stroke. These results suggest that the newsqol would be a good tool for carers to routinely use to evaluate the problems of patients and to explain and share information and care with them in a patient-centred approach, which is crucial among people with disabilities [23]. Maclean et al. observed that participation in rehabilitation of stroke patients was shaped by favourable attitudes, motivation, reassurance and provision of information [39]. Adherence to treatment is better among patients who perceive the therapeutic communication skill of their physician to be high [21]. Unfortunately, information and care services may not be provided equitably, leaving the people with socioeconomic difficulties with a lack of explanation, treatment, and treatment adherence [23].

Home-based healthcare requires stroke-survivors to find new ways to solve their problems within their families as well as with medico-social workers and others. We found that dissatisfaction with the possibility of receiving help when necessary was also associated with mobility and self-care. The mobility and self-care of patients may be better evaluated and appropriate technical and personal aids may be provided. The correlation revealed in our study between dissatisfaction with information about consequences/change over time of stroke and vision highlights that some subjects with visual impairment feared an aggravation of their problem and wanted it to be monitored. A weak association was observed between on one hand, being confident about where to get information about stroke, to look after other stroke-survivors or financial help, and being confident about whom to contact from community services if needed, and on the other hand, communication, feelings and interpersonal relationships. This information was thus perceived as less important than the information and home-care actually received. Home-care services can sustain stroke-survivors’ QoL by prolonging the ability to live independently at home which is closely linked to a positive sense of identity [38]. For these reasons, it is necessary to limit discrepancies between survivors’ and professionals’ views of need, bearing in mind that assessment processes favour the professionals’ point of view [49]. Stroke care that supports patients and caregivers by meeting their needs and demands could positively impact their QoL [50]. Therefore, improving home-care services provides an opportunity to interact allowing more effective collaborations between patients and medico-social workers. Professionals can also review their role and their practices in new and comprehensive perspectives [19]. Stroke-survivors and carers should redefine community interventions accordingly. Such approaches could improve the QoL of family-caregivers, which is highly associated with that of patients [22]. Our findings may help public policies aimed at improving professional practices, the quality of care and support, and patient QoL.

Our results further raise the question concerning the needs and satisfaction of stroke-survivors living at home in terms of information and care services as well as interactions with social and medical carers according to recovery stage and possible relapse over time. The changing nature of needs at different stages of recovery may not be paid sufficient attention. A telephone service could help. Use of IT technology has been proposed to promote person-centred rehabilitation [51]. It has been shown, with Telestroke, that videoconference calls can help to reduce stress, provide reassurance about the secondary effects of treatment, improve compliance with prescriptions, and yield valuable information about services [49]. A simple questionnaire measuring functional impairments, newsqol, and dissatisfaction with information and home-care services received/needed may then be administered via telephone to elucidate the situation and reveal changes in need over time.

### Strengths and limitations

This study is original in its two-stage specific recruitment process: identification of stroke-survivors based on a medico-administrative database, and then clinical confirmation of the diagnosis based on data from hospital medical records. Our choice to confirm all stroke diagnoses, 2 years after onset, was unusual, but was the only way to exclude pathologies that mimic stroke [52]. Such designs are rare because they are very expensive and it is difficult to organise a study 24 months after stroke onset. The participation rate estimate was low but similar to that in recent literature [53]. The relatively small sample is explained by the proportion of deaths two years after stroke and the limitation to survivors living at home (the study excluded those who were living in institutional settings, those who changed their residence, especially to live with their son or daughter, and those who failed to respond). Among stroke-survivors, aphasic patients were also included in the sample with appropriate interview training for researchers. Studying patients 2 years post-stroke creates an opportunity to provide valuable data about patients’ QoL and their information/care needs in the home-care system. The patient and his or her family may have adapted to their new situation, reorganised their daily lives, and become accustomed to caregiving [54]. Our results should be interpreted with caution for several reasons. We surveyed a small sample of volunteers who gave their consent. Requesting informed consent sent via the postal service may have reduced the response rate. Second, the interviews took place at patients’ homes, which involved intimacy and some people may find this difficult. Third, the participants may be more likely to be concerned by health issues, willing to share their opinions with us and happy to make their views broadly known.

## Conclusions

The present study among survivors living at home, two years after stroke onset, demonstrates that they suffered from a wide range of functional impairments which were associated with low quality of life in a number of domains, especially those reflecting depressive symptoms (pain, cognition, vision, communication, mental feelings, interpersonal relationships, emotion, sleep and fatigue) and also mobility and self-care. Many survivors had a low-level education, a lower income, and had left their job at stroke onset. Various domains of quality of life, especially those reflecting depressive symptoms, mobility and self-care were strongly associated with dissatisfaction with information about stroke and its consequences/change over time, accuracy of information received, help received, coordination between services, and possibility of receiving help when necessary. The newsqol is a good tool which deserves to be routinely used to measure quality of life, the recovery of patients, and their needs in terms of information, help and care. The depressive symptoms identified should be appropriately treated. Our findings may help public policies aimed at improving professional practice at patients’ homes, the quality of care and support, and the quality of life and mental status of stroke-survivors.

## Competing interests

The authors declare that they have no competing interests.

## Authors’ contributions

MB: conceived and carried out the study, and had the main responsibility for the study and writing the manuscript. ELB: realized the statistical analysis and participated in data analysis. KC: participated in data analysis and in writing the manuscript. NC: participated in data analysis and in writing the manuscript. All authors read and approved the final manuscript.

## Pre-publication history

The pre-publication history for this paper can be accessed here:

http://www.biomedcentral.com/1471-2377/14/92/prepub

## Supplementary Material

Additional file 1 Newcastle Stroke-Specific Quality of Life Measure (Newsqol) [7]. Click here for file

Additional file 2 Functional deficiencies of survivors: %. Click here for file

## References

[B1] Murray CJ Vos T Lozano R Naghavi M Flaxman AD Michaud C Ezzati M Shibuya K Salomon JA Abdalla S Aboyans V Abraham J Ackerman I Aggarwal R Ahn SY Ali MK Alvarado M Anderson HR Anderson LM Andrews KG Atkinson C Baddour LM Bahalim AN Barker-Collo S Barrero LH Bartels DH Basáñez MG Baxter A Bell ML Benjamin EJ Disability-adjusted Life Years (DALYs) for 291 Diseases and Injuries in 21 Regions, 1990-2010: a Systematic Analysis for the Global Burden of Disease Study 2010 Lancet 2012 380 9859 2197 2223 10.1016/S0140-6736(12)61689-4 23245608

[B2] Eurostats Health statistics – Atlas on mortality in the European Union. Theme: Population and social conditions Collection: statistical books 2009 Luxembourg: Office for Official Publications of the European Communities

[B3] OCDE Panorama de la santé 2013: Les indicateurs de l’OCDE 2013 OCDE http://dx.doi.org/10.1787/health_glance-2013-fr

[B4] Gokkaya NKO Aras MD Cakci A Health-related quality of life of Turkish stroke survivors Int J Rehabil Res 2005 28 3 229 235 10.1097/00004356-200509000-00005 16046916

[B5] Osberg JS DeJong G Haley SM Seward ML McGinnis GE Germaine J Predicting long-term outcome among post-rehabilitation stroke patients Am J Phys Med Rehabil 1988 67 94 103 10.1097/00002060-198806000-00002 3377892

[B6] Baumann M Lurbe-Puerto K Alzahouri K Aïach P Increased residual disability among post-stroke survivors, and the repercussions for the lives of informal caregivers Top Stroke Rehabil 2011 18 2 162 171 10.1310/tsr1802-162 21447466

[B7] Buck D Jacoby A Massey A Steen N Sharma A Ford GA Development and validation of NEWSQOL, the Newcastle Stroke-Specific Quality of Life Measure Cerebrovasc Dis 2004 17 2–3 143 152 14707414 10.1159/000075783

[B8] Duncan PW Jorgensen HS Wade DT Outcome measures in acute stroke trials: a systematic review and some recommendations to improve practice Stroke 2000 31 1429 1438 10.1161/01.str.31.6.1429 10835468

[B9] Ahmed S Schwartz C Ring L Sprangers MAG Applications of health-related quality of life for guiding health care: advances in response shift research J Clin Epidemiol 2009 62 11 1115 1117 10.1016/j.jclinepi.2009.04.006 19595571

[B10] Chau N Lemogne C Legleye S Choquet M Falissard B Fossati P Are occupational factors and mental difficulty associated with occupational injury? J Occup Environ Med 2011 53 1452 1459 10.1097/JOM.0b013e318237a14b 22076039

[B11] Lemogne C Niedhammer I Khlat M Ravaud JF Guillemin F Consoli SM Fossati P Chau N Gender differences in factors accounting for the association between self-reported depressive mood and premature mortality: A 12-year follow-up population-based study J Affect Disord 2012 136 267 275 10.1016/j.jad.2011.11.041 22197508

[B12] Kotila M Numminen H Waltimo O Kaste M Depression after stroke: result of the FINNSTROKE study Stroke 1998 29 368 372 10.1161/01.STR.29.2.368 9472876

[B13] Niedhammer I Bourgkard E Chau N Behavioural and occupational factors in the explanation of social inequalities in premature and total mortality: A 12.5-year follow-up study of the Lorhandicap survey Eur J Epidem 2011 26 1 12 10.1007/s10654-010-9506-9 PMC3515451 20845063

[B14] Kerr GD Slavin H Clark D Coupar F Langhorne P Stott DJ Do vascular risk factors explain the association between socioeconomic status and stroke incidence: a meta-analysis Cerebrovasc Dis 2011 31 1 57 63 10.1159/000320855 20980755

[B15] Ahmed A Lemkau JP Nealeigh N Mann B Barriers to healthcare access in a non-elderly urban poor American population Health Soc Care Community 2001 9 445 453 10.1046/j.1365-2524.2001.00318.x 11846824

[B16] European Foundation for the Improvement of Living and Working Conditions Evaluating the quality of society and public services 2010 Second European Quality of Live Survey http://www.eurofound.europa.eu/publications/

[B17] Hartman-Maeir A Soroker N Ring H Avni N Katz N Activities, participation and satisfaction one-year post stroke Disabil Rehab 2007 29 7 559 566 10.1080/09638280600924996 17453976

[B18] Owolabi MO What are the consistent predictors of generic and specific post-stroke health-related quality of life? Cerebrovasc Dis 2010 29 2 105 110 10.1159/000262305 19955733

[B19] de Weerd L Rutgers WAF Groenier KH van der Meer K Perceived wellbeing of patients one year post-stroke in general practice - recommendations for quality after care BMC Neurol 2011 11 42 53 10.1186/1471-2377-11-42 21453512 PMC3080803

[B20] EuroLIFE Quality of National Health Service 2007 Eurofound quality of life in Europe http://www.eurofound.europa.eu/areas/qualityoflife/eurlife/index.php?template=3&radioindic=15&idDomain=1

[B21] Baumann M Baumann C Le Bihan E Chau N How patients perceive the therapeutic communications skills of their general practitioners, and how that perception affects adherence: use of the TCom-skills GP scale in a specific geographical area BMC Health Serv Res 2008 8 244 256 10.1186/1472-6963-8-244 19046433 PMC2612661

[B22] Baumann M Couffignal S Le Bihan E Chau N Life satisfaction two-years after stroke onset: the effects of gender, occupational status, memory function and quality of life among stroke patients (Newsqol) and their family caregivers (Whoqol-bref) in Luxembourg BMC Neurol 2012 12 105 116 10.1186/1471-2377-12-105 23009364 PMC3551740

[B23] Iezzoni LI O’Day BL More than ramps. A Guide to improving health care quality and access for people with disabilities 2006 Oxford: Oxford University Press 366

[B24] Mold F McKevitt C Wolfe C A review and commentary of the social factors which influence stroke care: issues of inequalities in qualitative literature Health Soc Care Community 2003 11 5 405 414 10.1046/j.1365-2524.2003.00443.x 14498837

[B25] Cramm JM Strating MMH Nieboer AP Satisfaction with care as a quality-of-life predictor for stroke patients and their caregivers Qual Life Res 2012 21 1719 1725 10.1007/s11136-011-0107-1 22230965 PMC3496478

[B26] Baumann M Euller-Ziegler L Guillemin F Evaluation of the expectations osteoarthritis patients have concerning healthcare, and their implications for practitioners Clin Experim Rheum 2007 25 404 409 17631736

[B27] WHO Projections of mortality and burden of disease, 2004-2030 http://www.who.int/healthinfo/global_burden_disease/projections2004/en/

[B28] Wagner EH Meeting the needs of chronically ill people BMJ 2001 323 945 946 10.1136/bmj.323.7319.945 11679369 PMC1121493

[B29] STATEC Population et emploi/Etat de la population. Population par canton et commune 1821–2011 2008 http://www.statistiques.public.lu/stat/TableViewer/tableView.aspx?ReportId=397&IF_Language=fra&MainTheme=2&FldrName=1

[B30] Kelly-Hayes PM Robertson JT Broderick JP Duncan PW Hershey LA Roth EJ Thies WH Trombly CA The American Heart Association stroke outcome classification Stroke 1998 29 1274 1280 10.1161/01.STR.29.6.1274 9626308

[B31] Simon C Little P Birtwistle J Kendrick T A questionnaire to measure satisfaction with community services for informal carers of stroke patients: construction and initial piloting Health Soc Care Community 2003 11 2 129 137 10.1046/j.1365-2524.2003.00408.x 14629215

[B32] Simon C Kumar S Kendrick T Formal support of stroke survivors and their informal carers in the community: a cohort study Health Soc Care Community 2008 16 6 582 592 10.1111/j.1365-2524.2008.00782.x 18371168

[B33] Zhang X Bullard KM Cotch MF Wilson MR Rovner BW McGwin GJ Owsley C Barker L Crews JE Saaddine JB Association between depression and functional vision loss in persons 20 years of age or older in the United States NHANES 2005–2008 JAMA Ophthalmol 2013 131 5 573 581 10.1001/jamaophthalmol.2013.2597 23471505 PMC3772677

[B34] Gauchard GC Deviterne D Guillemin F Sanchez J Perrin P Mur JM Ravaud JF Chau N Prevalence of sensorial and cognitive disabilities and falls, and their relationships: A community-based study Neuroepidemiol 2006 26 108 118 10.1159/000090445 16374036

[B35] Cruice M Hill R Worrall L Hickson L Conceptualising quality of life for older people with aphasia Aphasiology 2010 24 3 327 347 10.1080/02687030802565849

[B36] Lynch EB Butt Z Heinemann A Victorson D Nowinski CJ Perez L Cella D A qualitative study of quality of life after stroke: The Importance of social relationships J Rehab Med 2008 40 7 518 523 10.2340/16501977-0203 PMC3869390 18758667

[B37] Pilkington FB A qualitative study of life after stroke J Neurosc Nurs 1999 31 6 336 347 10.1097/01376517-199912000-00004 10726242

[B38] Clarke P Black SE Quality of Life following stroke: Negotiating Disability, Identity, and Resources J Applied Gerontol 2005 24 4 319 336 10.1177/0733464805277976

[B39] Maclean N Pound P Wolfe C Rudd A Qualitative analysis of stroke patients’ motivation for rehabilitation BMJ 2000 321 1051 1054 10.1136/bmj.321.7268.1051 11053175 PMC27512

[B40] Lincoln NB Kneebone II Macniven JAB Morris RC Psychological Management of Stroke 2011 Milton, Queensland: John Wiley & Sons, Inc. 638p

[B41] Harvey PO Fossati P Pochon JB Levy R Lebastard G Lehéricy S Allilaire JF Dubois B Cognitive control and brain resources in major depression: an fMRI study using the n-back task Neuroimage 2005 26 860 869 10.1016/j.neuroimage.2005.02.048 15955496

[B42] Alonso J Codony M Kovess V Angermeyer MC Katz SJ Haro JM De Girolamo G De Graaf R Demyttenaere K Vilagut G Almansa J Lépine JP Brugha TS Population level of unmet need for mental healthcare in Europe Br J Psychiatry 2007 190 299 306 10.1192/bjp.bp.106.022004 17401035

[B43] Casacalenda N Perry JC Looper K Remission in major depressive disorder: a comparison of pharmacotherapy, psychotherapy, and control conditions Am J Psychiatry 2002 159 1354 1360 10.1176/appi.ajp.159.8.1354 12153828

[B44] Wang PS Lane M Olfson M Pincus HA Wells KB Kessler RC Twelve-month use of mental health services in the United States: results from the National Comorbidity Survey Replication Arch Gen Psychiatry 2005 62 629 640 10.1001/archpsyc.62.6.629 15939840

[B45] Briffault X Morvan Y Rouillon F Dardennes R Lamboy B [Use of services and treatment adequacy of major depressive episodes in France] Encéphale 2010 36 suppl 2 D48 D58 20513461 10.1016/j.encep.2008.10.011

[B46] Mar J Arrospide A Begiristain JM Larrañaga I Elosegui E Oliva-Moreno J The impact of acquired brain damage in terms of epidemiology, economics and loss in quality of life BMC Neurol 2011 11 46 57 10.1186/1471-2377-11-46 21496356 PMC3098775

[B47] Haacke C Althaus A Spottke A Siebert U Back T Dodel R Long-term outcome after stroke: evaluating health-related quality of life using utility measurements Stroke 2006 37 1 193 198 10.1161/01.STR.0000196990.69412.fb 16339458

[B48] The World Bank GDP per inhabitant 2011 http://donnees.banquemondiale.org/indicateur/ NY.GDP.PCAP.CD

[B49] Grant JS Elliott TR Weaver MM Bartolucci AA Giger JN Telephone intervention with family caregivers of stroke survivors after rehabilitation Stroke 2002 33 2060 2065 10.1161/01.STR.0000020711.38824.E3 12154263

[B50] Boter H de Haan RJ Rinkel GLE Clinimetric evaluation of a satisfaction with Stroke-Care questionnaire J Neurol 2003 250 534 541 10.1007/s00415-003-1031-2 12736731

[B51] Gzil F Lefeve C Cammelli M Pachoud B Ravaud JF Leplège A Why is rehabilitation not yet fully person-centred and should it be more person-centred? Disability Rehabil 2009 29 20–21 1616 1624 10.1080/09638280701618620 17922330

[B52] National Stroke Association Hope: The stroke recovery guide 2007 Denver Colorado: The National Stroke Association

[B53] Bergstöm AL Eriksson G von Koch L Kerstin T Combined life satisfaction with stroke and their caregivers: associations with caregiver burden and the impact of stroke Health Qual Life Out 2011 9 1 10 10.1186/1477-7525-9-1 PMC3024212 21223594

[B54] Ostwald SK Godwin KM Cron SG Predictors of life satisfaction in stroke survivors and spousal caregivers twelve to twenty-four months post discharge from inpatient rehabilitation Rehabil Nurs 2009 34 4 160 174 19583057 10.1002/j.2048-7940.2009.tb00272.x PMC2771652

