# Supplementary material for: Associations between quality of life and socioeconomic factors, functional impairments and dissatisfaction with received information and home-care services among survivors living at home two years after stroke onset
Source: BMC Neurol. 2014 Apr 28;14:92. doi: 10.1186/1471-2377-14-92 (PMC4021376; doi:10.1186/1471-2377-14-92)
Supplement: Additional file 1 — Newcastle Stroke-Specific Quality of Life Measure (Newsqol) [7]. [file 1471-2377-14-92-S1.doc]

**Additional file 1 Newcastle Stroke-Specific Quality of Life Measure (Newsqol) [7].**

| **Mobility**  1. Do you get around in a wheelchair because of the stroke? |
| --- |
| 2. Because of the stroke, do you have any difficulty walking half a mile? |
| 3. Because of the stroke, do you have any difficulty walking up or down hills? |
| 4. Because of the stroke, do you walk with a stick or frame or by holding onto things? |
| 5. Do you feel as if you walk slowly because of the stroke? |
| 6. Do you have difficulty managing stairs on your own because of the stroke? |
| 7. Do you have difficulty bending down because of the stroke? |
| 8. Do you find that you are unsteady on your feet because of the stroke? |
| 9. Because of the stroke, do you have difficulty standing for any length of time? |
| **Self-care**  10. Do you have difficulty with housework because of the stroke? |
| 11. Because of the stroke, do you have difficulty with cooking? |
| 12. Because of the stroke, do you have difficulty preparing food, for example cutting a slice of bread or cutting up vegetables? |
| 13. Do you have difficulty managing the shopping because of the stroke? |
| 14. Because of the stroke, do you have difficulty using public transport? |
| 15. Do you have difficulty getting washed by yourself because of the stroke? |
| 16. Because of the stroke, do you have difficulty getting dressed by yourself, including things like zips and buttons? |
| 17. Do you have difficulty getting in/out of the bath or shower on your own because of the stroke? |
| **Pain / sensory**  18. Do you have any pain because of the stroke? |
| 19. How often do you have pain because of the stroke? |
| 20. Because of the stroke, do you have difficulty picking up small things? |
| **Vision**  21. Do you have problems with your eyesight because of the stroke? |
| 22. Do you have any difficulty with reading because of your eyesight (because of the stroke)? |
| **Cognition**  25. Do you find it difficult to concentrate for long because of the stroke? |
| 24. Because of the stroke, are there times when you forget what you have said or what people say to you? |
| 23. Because of the stroke do you find it difficult to solve problems or make decisions? |
| 26. Would you say you keep forgetting things because of the stroke? |
| 27. Because of the stroke, do you find it difficult to think clearly? |
| **Communication**  28. Do you feel as though your speech is slurred at all because of the stroke? |
| 29. Do you find it difficult to make yourself understood because of the stroke? |
| 30. Because of the stroke, are there times when you have difficulty expressing yourself? |
| 31. Do you have any difficulty with writing because of the stroke? |
| **Feelings**  32. Do you feel less independent than you were, because of the stroke? |
| 33. Has the stroke changed the way you feel about yourself? |
| 34. To what extent would you say your life has changed because of the stroke? |
| 35. Do you feel depressed because of the stroke? |
| 36. Does the stroke make you feel useless at all? |
| 37. Do you feel you have less control over what is happening in your life because of the stroke? |
| **Interpersonal relationships**  38. Because of the stroke, do you argue more with close friends or family? |
| 39. Has the stroke put any strain on your relationship with your spouse or partner? |
| 40. Does the stroke interfere with your sex life and if so, how much? |
| 41. Are you short-tempered because of the stroke? |
| 42. Are you less tolerant because of the stroke? |
| 43. Because of the stroke, have you become nervous about meeting people? |
| **Emotion**  44. Do you get more emotional because of the stroke? |
| 45. Do you sometimes cry at the least thing because of the stroke? |
| 46. Are you worried that you could have another stroke? |
| 47. Because of the stroke, do you worry about becoming dependent on other people? |
| **Sleep**  48. Do you have problems sleeping at night because of the stroke? |
| 49. Do you have difficulty getting off to sleep because of the stroke? |
| 50. Because of the stroke, do you sometimes wake up too early? |
| 51. Do you find you need a lot of rest because of the stroke? |
| 52. Do you ever feel exhausted because of the stroke? |
| 53. Do you feel that you lack energy because of the stroke? |
| **Fatigue**  54. Because of the stroke, are there days when you feel you could sleep all the time? |
| 55. Do you doze off during the day because of the stroke? |
| 56. Because of the stroke, do you feel that you can't be bothered with things at times? |

### The response, for each item of each domain, ranged from 1 (worst) to 4 (best). The score of each domain was defined as the sum of the responses, and then changed to range from 0 (lowest) to 100 (best).
