# Supplementary material for: Associations between quality of life and socioeconomic factors, functional impairments and dissatisfaction with received information and home-care services among survivors living at home two years after stroke onset
Source: BMC Neurol. 2014 Apr 28;14:92. doi: 10.1186/1471-2377-14-92 (PMC4021376; doi:10.1186/1471-2377-14-92)
Supplement: Additional file 2 — Functional deficiencies of survivors: %. [file 1471-2377-14-92-S2.doc]

**Additional file 2 Functional deficiencies of survivors: %.**

|  | **%** | **AHA.SOC domains** |
| --- | --- | --- |
| **Motor deficiencies** |  |  |
| Tetraplegic (4 members) | 2.1 | Motor |
| Hemiplegic (2 members on the same side) | 10.6 | Motor |
| Only one upper limb or lower | 3.2 | Motor |
| Facial paralysis | 6.4 | Motor |
| Other motor deficiencies | 16.0 | Motor |
| **Visual deficiencies** |  |  |
| Loss of half of the visual field | 12.8 | Vision |
| Other disturbances (visual field, colour) | 7.4 | Vision |
| **Auditory deficiencies** |  |  |
| Disorders of hearing (loss, reduction) | 9.6 | Sensory |
| **Sensory deficiencies** |  |  |
| Sensory deficiencies (numbness, hot-cold, touch) | 25.5 | Sensory |
| Loss of taste | 9.6 | Sensory |
| Pain | 23.4 | Sensory |
| **Deficiencies of language and communication** |  |  |
| Total loss of language | 3.2 | Motor |
| Partial loss of language | 2.1 | Language |
| Temporary loss of language | 3.2 | Language |
| Inversion of words | 9.6 | Language |
| Disorders of the voice | 9.6 | Language |
| Disorders of comprehension | 16.0 | Language |
| Partial or total disorder of reading and/or writing | 19.1 | Language |
| **Organ deficiencies** |  |  |
| Urinary incontinence | 10.6 | Incontinence |
| Faecal incontinence | 5.3 | Incontinence |
| Swallowing disorders | 18.1 | Motor |
| **Other deficiencies** |  |  |
| Disorders of memory | 31.9 | Cognition |
| Personality disorders | 17.0 | Affect |
